# The potential role of preventive and therapeutic immunonutrition strategies for pediatric food allergy: A mini-review

**DOI:** 10.3389/fnut.2022.1050554

**Published:** 2022-12-02

**Authors:** Serena Coppola, Laura Carucci, Roberta De Michele, Roberto Berni Canani

**Affiliations:** ^1^Department of Translational Medical Science, University of Naples Federico II, Naples, Italy; ^2^ImmunoNutritionLAB at CEINGE Advanced Biotechnologies, University of Naples Federico II, Naples, Italy; ^3^Department of Community Medicine and Primary Care, University of Naples Federico II, Naples, Italy

**Keywords:** pediatric food allergy, allergic diseases, gut microbiome, active diet, immunonutrition, allergy

## Abstract

Food allergy (FA) represents one of the main chronic conditions of the pediatric population. The gut microbiome (GM)-immune system axis is a milestone in affecting FA susceptibility. The dynamic and bidirectional crosstalk between the GM and immune system starts early in life, and it is deeply modulated during the first 1,000 days of life. Nutritional factors during this crucial period mainly influence the proper GM-immune system development and function across the lifespan, with potential beneficial or detrimental effects on health status. Immunonutrition strategies, applied from conception, could represent an innovative target for prevention and treatment of pediatric FA. Here we described the potential role of preventive and therapeutic immunonutrition strategies for pediatric FA, highlighting putative future perspectives in this field.

## Introduction

**Figure 1:**
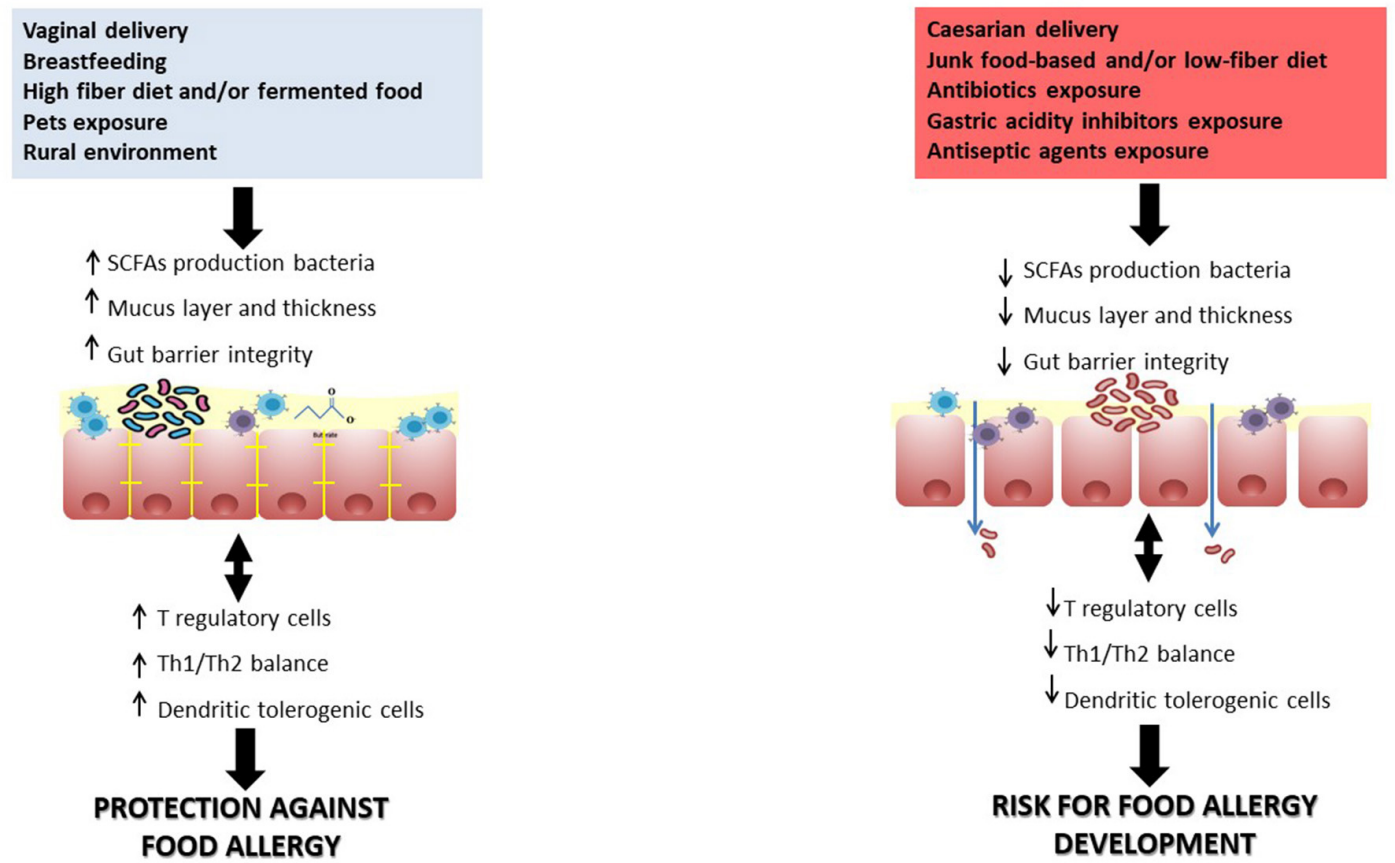
The GM-immune system axis is responsible for pivotal regulation of long term health status, mainly through epigenetic mechanisms. For instance, healthy dietary and lifestyle habits such as Mediterranen diet (full of fibers, fermented foods, antioxidants, omega-3), breastfeeding, exposure to pets and/or rural environment, vaginal delivery, and use of probiotics are responsible for a positive influence on the axis. Conversely, cesarean section, prenatal, and early-life exposure to antibiotics/gastric acidity inhibitors/antiseptic agents, unhealthy diet (low fibers/high saturated fats and junk foods) influence negatively the axis. These environmental variables are becoming important targets for active prevention of FA.

Food allergy (FA) is one of the major chronic diseases of the pediatric population, affecting up to 10% of children in industrialized countries, mainly in the first years of life (1). Pediatric FA derives from a failure of the immune tolerance network in early life. Alterations of the proper development and functioning of the immune system, leading to the immune tolerance network failure, are mainly modulated by the gut microbiome (GM). This evidence suggests the importance of the “GM-immune system axis” (2, 3). Indeed, negative factors affecting GM composition in the pre-natal or early life (e.g., cesarean delivery, proton pump inhibitor, anti-septic and antibiotic use etc.) leads to a reduction in bacterial biodiversity, to an increased intestinal permeability, and to an impairment of GM function with a decreased production of immunomodulatory compounds such as short chain fatty acids (SCFAs), deeply involved in the regulation of immune tolerance mechanisms and T regulatory (T reg) induction (4, 5). Indeed, FA children present specific GM signatures and growing evidence shows that GM alterations, leading to an impairment of the immune system, could also facilitate the occurrence of other chronic non-communicable diseases (NCDs), such as obesity and autoimmune and inflammatory disorders (5–7). By contrast, exposure to factors that positively affect the structure and function of the GM (e.g., breastfeeding, high fiber diet, assumption of pre-, pro, and synbiotics, etc.) leads to a positive modulation of the axis and potential subsequent protection against the occurrence of NCDs in early life as well in adulthood (8–10). The GM of healthy breastfed infants is mainly characterized by an abundance of *Bifidobacteria*, which play a pivotal role in optimal immune system development. Indeed, *Bifidobacterium* species through the production of some immunomodulating factors such as SCFAs are able to exert a positive influence on innate immunity, secretory IgA production and Th1/Th2 balance, favoring a Th1 response with a protective effect against allergic diseases. The underlying mechanism is not completely defined, but it involves an increase of regulatory T cells, needed for maintaining the intestinal immune homeostasis (5, 11–14). Figure 1 graphically represents the mechanisms involved in GM-immune system axis regulation. The dynamic and bidirectional interaction between the GM and immune system starts early in life, from the conception and has lifelong effects. However, the crucial time frame to establish the health status for the baby and for his adult life are the “first 1,000 days” (15), a critical window of opportunity in which both GM colonization and immune system development occur (16).

Starting from this evidence, an early integrated approach could prevent and counteract gut dysbiosis by modulating the interaction among dietary factors, the GM, and the immune system and it open the way for the “immunonutrition” concept. This strategy refers to the potential of specific nutrients in the modulation of immune system activity (17). From this innovative point of view, the immunonutrition approach, applied early in life, could represent a new strategy to prevent and to potentially treat GM-immune system derived diseases such as FA.

In this Mini-Review we described the preventive and potential therapeutic immunonutrition strategies for pediatric FA highlighting future perspectives in this field.

## The main immunonutrition strategies to prevent FA

During the critical period of the first 1,000 days of life, nutritional factors are the main environmental factors influencing the immune system function and the future disease susceptibility (18). In this paragraph we report the main immunonutrition strategies to prevent FA in the first 1,000 days.

The first nutritional exposure for the fetus derives from the maternal diet during pregnancy, representing one of the main *in utero* programming factors able to modulate the risk of later life allergy (19). The consumption, during pregnancy, of a holistic diet based on healthy foodstuffs typical of the Mediterranean diet and low in saturated fats and refined foods, may protect the offspring against allergies (20–25). The Mediterranean diet is characterized by high consumption of unprocessed cereals, vegetables, legumes, fruits, nuts, and fish, providing large amounts of poly-unsaturated (eicosapentaenoic and docosahexaenoic acids) and mono-unsaturated fatty acids, vitamins, trace elements, polyphenols, and antioxidants, known for their anti-inflammatory and anti-allergic properties (26). Notably, the Mediterranean diet's beneficial effects are due to the interactive and synergistic nutrient combinations, and the epigenetic changes that affect the gene expression is one of the mechanisms by which this dietary pattern could lead to regulatory effects in the offspring immune system (27).

After delivery, maternal diet remains one of the main factors able to influence the allergy risk in the newborn through breastfeeding. A high adherence to the Mediterranean diet during lactation positively influences the composition of human milk (HM), potentially protecting the child against FA (28). Although the European Academy of Allergy and Clinical Immunology (EAACI) guidelines for the prevention of FA reports no recommendation for the use of breastfeeding to prevent FA, healthcare professionals should encourage breastfeeding wherever possible for its overall positive benefits for infants and mothers (29). HM is considered the “gold standard” for infant nutrition, and it is recommended to be the exclusive source of nourishment for the first 4–6 months of life and, after the start of complementary feeding at 4–6 months, up to 2 years of age or beyond (30). Several reasons explain the potential protective effects of HM on allergy susceptibility in children: breastfeeding extends the relationship with the maternal immune system, it could impact the immune tolerance to dietary antigens, and it is a source of a large amount of biologically active compounds that positively influence the infant's GM and immune system (31). Among the bioactive factors, breast milk contains oligosaccharides (HMOs), which have prebiotic activity, pass undigested to the colon, and serve as fermentation substrates for beneficial commensal bacteria, such as *Bifidobacterium* and *Lactobacillus*, to support the proper development of the GM (32). The main microbial fermentation products of HMOs are SCFAs, which exert immunomodulatory functions, beneficially modulating the immune system development and function (33). Among SCFAs, butyrate is able to modulate several tolerogenic mechanisms, exerting a pivotal role in the FA protection. In addition, considerable amounts of butyrate were detected in HM, confirming again the protective role of breastfeeding against pediatric FA (34). Furthermore, HM is a source of several non-nutritive protective factors, which provide passive immunity as well as stimulation for the maturation of the infant's GM and immune system through the interaction with the digestive and respiratory tracts mucous membranes, such as immunoglobulins (secretory IgA, IgM), cytokines, enzymes (lactoferrin, lysozyme, etc.), nucleotides, complement system components, leukocytes, microRNA and hormones (35).

The timing of the introduction of allergenic foods into the infant's diet is currently one of the most promising strategies to prevent FA. The current guidelines do not recommend avoiding or delaying the introduction of food allergens in the infant's diet to prevent allergy, suggesting the protective effect of early introduction of complementary foods against FA (29). The introduction of complementary solid foods and liquids, other than HM or infant formula, should not be started before 4 months and not be delayed beyond 6 months of life (36). At weaning, the complementary diet composition and the quality of foods are the major determinants of the GM development and function (37). The consumption in the first year of life of healthy foodstuffs such as fruit, vegetables, yogurt, and fish, has been associated with high levels of fecal butyrate and a reduced risk of FA (38). On the contrary, the increased consumption of ultra-processed foods typical of a Western diet, rich in dietary advanced glycation end-products (e.g., fast foods, roasted/barbecued meats, sweets, and beverages), could result in the development of allergic phenotypes (39).

Starting from all of this evidence, early immunonutrition strategies are the key to prevent the onset of FA and to protect against the future disease susceptibility.

## The potential therapeutic immunonutrition strategies for pediatric FA: The active diet therapy

In this paragraph, we focus on the potential therapeutic effects of immunonutrition against pediatric FA. Once FA have already arisen, the cornerstone of immunonutrition is moving from a passive line of action, based on the elimination from the diet of food allergens to relieve symptoms, to a “pro-active” one, underlining the possibility of positively modulating the immune system toward the immune tolerance acquisition. Different active dietary strategies could be adopted, based on a positive modulation of the GM-immune system axis. These could be summarized in the infant formula choice for infants who are not breastfed and are affected by one of the most common FA, the cow's milk protein allergy (CMA), and the use of pro-, pre-, and synbiotics, the dietary intake of baked foodstuffs containing the food allergens, and oral immunotherapy (17).

In CMA babies, if breastfeeding is not available, a substitutive infant formula adapted to CMA dietary management is required (40). The commercially available infant formulas for CMA management are extensively hydrolyzed whey formula, extensively hydrolyzed casein formula, soy formula, partially hydrolyzed rice formula, and amino acid-based formula (41). Substitutive formulas represent the main cost for the management of CMA, thus, options to speed up the immune tolerance acquisition are important for families of affected infants and for health care systems. Our Research Team have recently demonstrated an *in vitro* different modulation exerted by the protein fraction of the above-mentioned infant formulas used for CMA management on tolerogenic mechanisms (42). In particular, we showed that only the protein fraction deriving from the extensively hydrolyzed casein formula stimulated tolerogenic effects, at least by an epigenetic modulation of the gene expression of FoxP3, which plays a central role in maintaining the homeostasis and tolerance of the immune system (42). Similarly, several randomized-controlled clinical trials have demonstrated that the consumption of extensively hydrolyzed casein formula (alone or added with the probiotic *Lactobacillus rhamnosus* GG), compared to other substitutive formulas, could accelerate the acquirement of the immune tolerance and could protect CMA children against the onset of Atopic March (43–45).

Furthermore, factors that positively modulate the composition and function of the GM, e.g., pro-, pre, and synbiotics, could drive the immune system of FA children toward a tolerogenic pathway. The administration of probiotics (live microorganisms that, when administered in adequate amounts, confer health benefits) could be a useful strategy to improve gastrointestinal symptoms of CMA infants, as shown in a randomized study evaluating the efficacy of 1 × 10^9^ CFU daily dose of *Bifidobacterium lactis BB-12* and of 1 × 10^8^ CFU daily dose of *Streptococcus thermophilus TH-4* (46). Another double blind, randomized, placebo-controlled study, performed on 100 infants affected by CMA, indicated the efficacy of the oral administration of *Lactobacillus rhamnosus* GG in association with a cow's milk protein free-diet for 4 weeks in ameliorating symptoms such as vomiting, diarrhea, restiveness, bloody and mucous stool, and abdominal distension, compared to the placebo (47). Our group have also demonstrated that the consumption of an extensively hydrolyzed casein formula with added *Lactobacillus rhamnosus* GG determined a higher rate of immune tolerance acquirement and a lower incidence of other atopic manifestations onset at 6 months and 1 and 3 years later than the extensively hydrolyzed casein formula alone and the other commercially available substitutive formula for CMA management (43, 44, 48). Furthermore, our group have also shown that the management of CMA infants with an extensively hydrolyzed casein formula with added *Lactobacillus rhamnosus* GG resulted in an increase of the gut bacterial strains butyrate producing the main tolerogenic metabolite (49).

In addition, the role of prebiotics and synbiotics in the GM-immune system axis modulation has also been suggested. Prebiotics are the main substrate for the growth and/or functionality of GM beneficial microbes (50). The addition of the prebiotic lactose to an extensively hydrolyzed whey formula was able to increase the total fecal amounts of *Lactobacillus/Bifidobacteria* in CMA infants, resulting in an increase of the beneficial metabolite butyrate (51). Instead, synbiotics are defined as a mixture of live microorganisms and substrate(s) selectively used by host microorganisms that confer health benefits (52). A recent multicenter trial in which infants affected by non-IgE-mediated CMA were treated with amino acid-based formula containing a symbiotic, consisting of *Bifidobacterium breve M-16V* and fructo-oligosaccharides, elicited beneficial effects on the GM composition of CMA patients, bringing it close to a healthy breastfed GM (53, 54).

In weaned FA children, other active dietary strategies could be adopted, such as the possibility to introduce baked products containing the food allergens. In fact, the allergenic properties of food allergen proteins could be modified during food handling such as lactic fermentation and heat treatment (55), and the immunoglobulins-binding capability could be affected by these proteins' structural changes (glycation, aggregation, unfolding, etc…), with a potential decrease of allergenicity (56, 57). Furthermore, the exposure of food allergen proteins to elevated temperatures in culinary recipes in association with a matrix (e.g., wheat, sugar, and oil, for the creation of a muffin) resulted in a final reduced immunoreactivity if compared with just heated food containing allergens (58). It has been reported that there is a generally good prognosis of cow's milk and egg protein allergies with the introduction into the diet of particularly baked forms of milk and egg (55, 59–63); conversely, the prognoses of wheat and soy allergies have not been well describrd. Identifying children who tolerate baked products could be extremely important, both to increase the diet variety and the quality of life, but also to accelerate the immune tolerance acquisition (64). In this direction, due to the large number of patients tolerant to baked products, a step-by-step approach has been proposed by the British Society for Allergy and Clinical Immunology. They have suggested “milk ladder” and “egg ladder” strategies, based on the gradual increase of quantity and allergenicity consumption of food allergens, to speed up the immune tolerance acquisition to fresh milk and egg (65). Unfortunately, to date, there are no diagnostic screening tests to detect patients that could tolerate baked products, and a need for standardization is required to use ladders safely and successfully in the clinical practice (66).

In the case of persistent FA, one of the potential therapeutic immunonutrition strategies is the oral immunotherapy (OIT). This kind of strategy consists of a regular administration of incremental doses of the culprit food, mainly adopted for peanuts, cow's milk, and eggs allergies (67–69). There are no standardized OIT protocols, but regular step-up consumption of the allergen has been shown to increase the reactivity threshold of a specific antigen, resulting in a protection against allergic reactions due to accidental ingestion of the culprit food (70). One of the weaknesses of OIT is related to the risk of the development of severe allergic reactions during the protocol as well as the lack of data regarding a long-term effect of this strategy (71). Indeed, OIT is able to transiently modulate different humoral and cellular pathways, but the ability to induce a long-lasting effect in terms of immune tolerance has not yet been established (17). OIT surely represents an emerging area of research, but due the limitations regarding safety and long-term efficacy, is not yet used in the clinical treatment routine (72). The development of biological therapies could change the fate of OIT. In fact different biological drugs administered as adjunctive OIT treatment have shown promising results in terms of OIT efficacy and safety (73).

## Discussion

The incidence and prevalence of pediatric FA has increased significantly in recent decades. Preventive and therapeutic strategies are advocated to limit the disease burden.

Through the immunonutrition approach, i.e., the ability of specific dietary factors to modulate the development and function of the immune system, pediatric FA could be prevented and, when already arisen, could be managed through an integrated proactive approach able to speed up the oral tolerance acquisition and to prevent the Atopic March onset.

**Figure 2:**
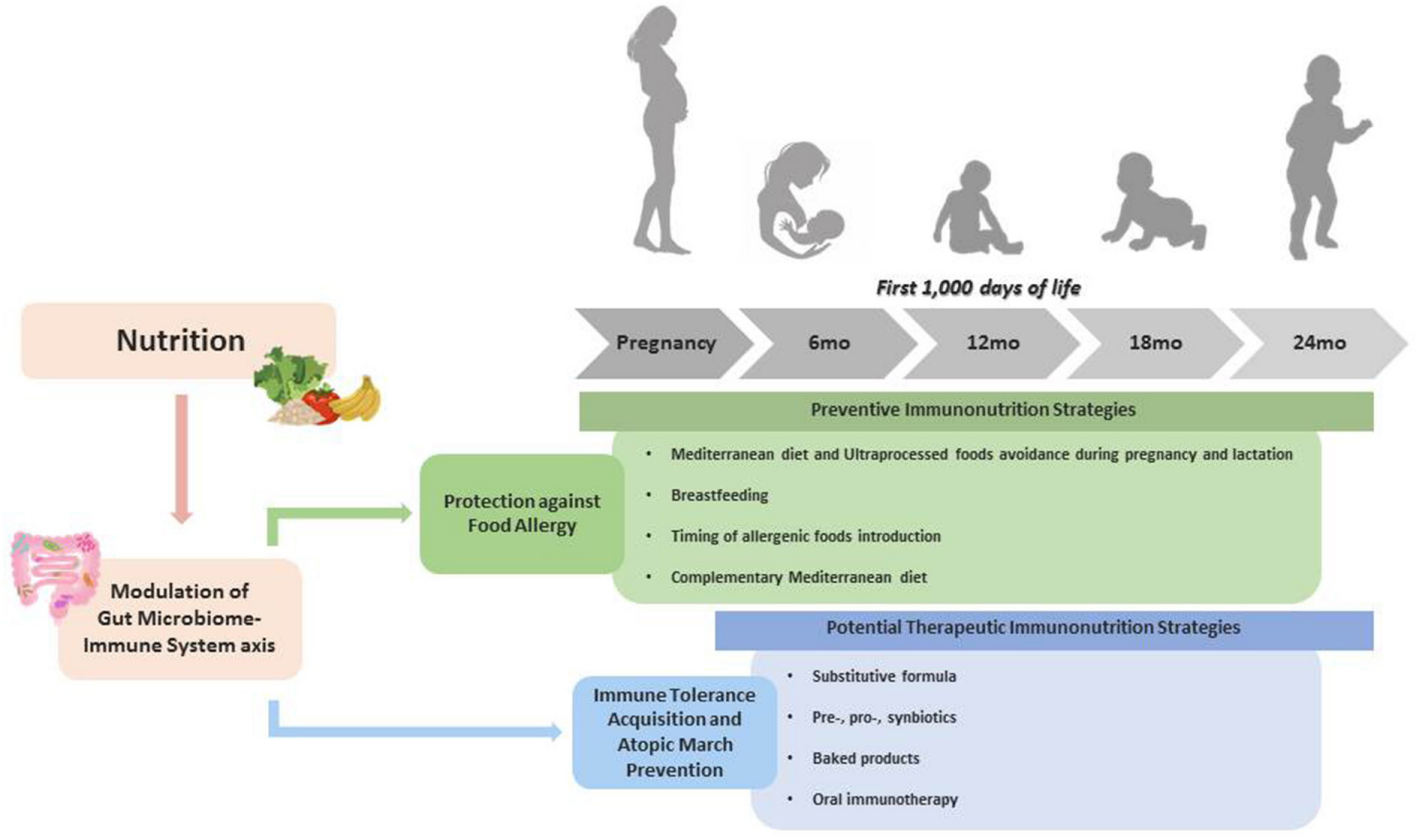
Through a positive modulation of GM-Immune System axis, nutritional factors are able to prevent and potentially treat FA.

This narrative Mini-Review provided an overview of the more solid and scientific-based preventive and therapeutic immunonutrition strategies for pediatric FA. Figure 2 graphically summarizes the key messages of these immunonutrition strategies.

## Author contributions

SC, LC, RDM, and RBC analyzed the literature and wrote and read the manuscript. All authors have substantially contributed to the development, conception, and design of the present Mini-Review and listed have made a direct and intellectual contribution to the manuscript and approved it for publication.

## Conflict of interest

The authors declare that the research was conducted in the absence of any commercial or financial relationships that could be construed as a potential conflict of interest.

## Publisher's note

All claims expressed in this article are solely those of the authors and do not necessarily represent those of their affiliated organizations, or those of the publisher, the editors and the reviewers. Any product that may be evaluated in this article, or claim that may be made by its manufacturer, is not guaranteed or endorsed by the publisher.
